# Implementation of an interactive mobile application to pilot a rapid assay to detect HIV drug resistance mutations in Kenya

**DOI:** 10.1371/journal.pgph.0000185

**Published:** 2022-02-16

**Authors:** Justin D. Vrana, Nuttada Panpradist, Nikki Higa, Daisy Ko, Parker Ruth, Ruth Kanthula, James J. Lai, Yaoyu Yang, Samar R. Sakr, Bhavna Chohan, Michael H. Chung, Lisa M. Frenkel, Barry R. Lutz, Eric Klavins, Ingrid A. Beck

**Affiliations:** 1 Department of Bioengineering, University of Washington, Seattle, Washington, United States of America; 2 Global Health of Women, Adolescents, and Children (Global WACh), School of Public Health, University of Washington, Seattle, Washington, United States of America; 3 Center for Global Infectious Disease Research, Seattle Children’s Research Institute, Seattle, Washington, United States of America; 4 Paul G. Allen Center for Computer Science & Engineering, University of Washington, Seattle, Washington, United States of America; 5 Department of Electrical and Computer Engineering, University of Washington, Seattle, Washington, United States of America; 6 Coptic Hope Center for Infectious Diseases, Nairobi, Kenya; 7 Center for Virus Research, Kenya Medical Research Institute, Nairobi, Kenya; 8 Department of Global Health, University of Washington, Seattle, Washington, United States of America; 9 Department of Medicine, Emory University, Atlanta, Georgia, United States of America; 10 Departments of Global Health, Medicine, Pediatrics, and Laboratory Medicine, University of Washington, Seattle, Washington, United States of America; National Institute of Neuroscience Department of Mental Retardation and Birth Defect Research: Kokuritsu Kenkyu Kaihatsu Hojin Seishin Shinkei Iryo Kenkyu Center Shinkei Kenkyujo Shippei Kenkyu Dainibu, BANGLADESH

## Abstract

Usability is an overlooked aspect of implementing lab-based assays, particularly novel assays in low-resource-settings. Esoteric instructions can lead to irreproducible test results and patient harm. To address these issues, we developed a software application based on “Aquarium”, a laboratory-operating system run on a computer tablet that provides step-by-step digital interactive instructions, protocol management, and sample tracking. Aquarium was paired with a near point-of-care HIV drug resistance test, “OLA-Simple”, that detects mutations associated with virologic failure. In this observational study we evaluated the performance of Aquarium in guiding untrained users through the multi-step laboratory protocol with little supervision. To evaluate the training by Aquarium software we conducted a feasibility study in a laboratory at Coptic Hope Center in Nairobi, Kenya. Twelve volunteers who were unfamiliar with the kit performed the test on blinded samples (2 blood specimens; 5 codons/sample). Steps guided by Aquarium included: CD4+ T-Cell separation, PCR, ligation, detection, and interpretation of test results. Participants filled out a short survey regarding their demographics and experience with the software and kit. None of the laboratory technicians had prior experience performing CD4+ separation and 7/12 had no experience performing laboratory-based molecular assays. 12/12 isolated CD4+ T cells from whole blood with yields comparable to isolations performed by trained personnel. The OLA-Simple workflow was completed by all, with genotyping results interpreted correctly by unaided-eye in 108/120 (90%) and by software in 116/120 (97%) of codons analyzed. In the surveys, participants favorably assessed the use of software guidance. The Aquarium digital instructions enabled first-time users in Kenya to complete the OLA-simple kit workflow with minimal training. Aquarium could increase the accessibility of laboratory assays in low-resource-settings and potentially standardize implementation of clinical laboratory tests.

## Introduction

In resource-rich communities, automation drastically improves the daily operation of clinical laboratories [1–3]. Patient samples can be quickly shipped to centralized laboratories for batch-processing and testing using highly efficient workflows that generate high-quality results while reducing costs and turnaround time [4, 5]. However, total automation is ill-suited for low-resource settings for many reasons. First, shipping of samples to centralized laboratories can take ≥10 days [6], which undermines the benefits of fast turnaround results from an automated workflow. Second, in small communities, the demand of a clinical assay may be low, requiring a longer waiting period to receive enough samples to complete a full batch. Finally, automation is often used in conjunction with high-throughput robotic equipment that is cost-prohibitive for small laboratories. In low-resource settings, high-quality and fast laboratory results for complex assays will likely require unorthodox approaches to build on low-cost equipment and small batches of samples.

HIV infects nearly 40 million people globally [7] and successful management of the infection relies on multiple laboratory tests. Recent advances include point-of-care HIV diagnosis and viral load quantification [8, 9]. Due to the complexity of HIV drug resistance (**HIVDR**) tests used to guide treatment regimens, these are performed in centralized, highly equipped laboratories [10–13]. In low-resource countries with high HIV prevalence like Kenya, few laboratories have the capacity to test for HIVDR [14]. For laboratories without access to sequencers, an oligonucleotide ligation assay (**OLA**) has been implemented [15] but onboarding OLA required extensive training due to its complexity.

We envision the use of software to automate a simplified version of OLA that uses low-cost equipment. To that end, we developed “OLA-Simple” which uses lyophilized reagents to simplify the workflow and lateral flow tests to provide visual results [16, 17]. We also developed a software application based on “Aquarium” [18] that employs human-in-the-loop automation to tightly integrate all the steps in OLA-Simple. Aquarium provides step-by-step interactive digital instructions, protocol management, data collection and sample tracking. In a pilot study at the University of Washington, Aquarium enabled minimally-trained students to accurately perform the OLA-Simple workflow [19]. Here, we demonstrate the use of the Aquarium-enabled HIVDR test in a small laboratory in Nairobi, Kenya.

## Methods

### Study design

A Seattle team travelled to Nairobi, Kenya to set-up a testing site at the Coptic Hope Center for Infectious Diseases, a large-scale antiretroviral treatment site [20], to evaluate the utility and performance of Aquarium in guiding first-time users to perform the OLA-Simple kits. We recruited 12 laboratory technicians from the Coptic Hospital clinical laboratories to perform OLA-Simple from April 4–13, 2018. We estimated that a sample size of 12 technicians, each testing ten HIVDR codons, was adequate to assess test feasibility and performance and to obtain representative feedback on the participants’ perceptions related to the OLA-Simple kit and Aquarium digital instructions. This study was approved by the Institutional Ethics Review Committee (IERC) of the Aga Khan University in Kenya, and Seattle Children’s Research Institute’s IRB. All participants provided written informed consent.

### Laboratory setup

The laboratory’s existing thermal cycler, biological safety hood, bench space, and refrigerator were utilized. The Seattle team brought the OLA-Simple kits, a minicentrifuge, micropipettes, scanner (CanoScan LiDE 300), tablets (Fire HD), foot controller, UPS battery backup and surge protector (APC 1500VA Compact), and server (Intel NuC NUC7i3BNH Mini PC/HTPC) to set up and run Aquarium. Aquarium code is publicly available [21].

### Evaluation of OLA-Simple

Two technicians worked with the Seattle team each day, with all 12 completing the project over a period of six days. Each participating technician completed a demographic questionnaire and were given a 30-minute introduction to the principles and procedures used in the kit. Then each processed and tested two blinded blood samples. CD4+ cells were separated from 0.5 mL uninfected blood, lysed, and then spiked with mixtures of plasmids containing known HIVDR mutations. After PCR amplification of a region of HIV *pol*, mutation-specific probes were annealed, ligated and then the ligated products detected using lateral flow strips. The lateral flow strips were scanned, and the images displayed on the tablets were used by the participants to make visual calls and generate a report using Aquarium. Finally, the participants completed a questionnaire to give feedback on their experience with the kits and software.

### Preparation of OLA-Simple kits

The OLA-Simple kit was prepared and assembled as previously described [19]. The EasySep CD4+ T-Cell isolation kit (STEMCELL Technologies, Vancouver, CA) was adapted to small blood volume processing, aliquoted and packaged in foil pouches. Reagents for PCR, ligation for detection of five HIV major NNRTI/NRTI resistance codons (K65R, K103N, Y181C, M184, and G190A) and lateral flow strips to detect ligation products were packaged in foil pouches with desiccant. Each kit component was labeled with a unique identifier, matching the images illustrated in Aquarium instructions.

### Post-analysis of samples in Seattle

The DNA yield in lysed cells obtained by Kenyan participants was assessed in Seattle by qPCR of human beta globin [22]. The lateral flow images and Aquarium reports generated by each participant were used to assess assay performance and interpretation of visual results. Test accuracy was determined by comparison to the expected genotype at each codon analyzed. Lateral flow strips images were re-analyzed using an in-house Python script [19] to determine if automated analyses improved test accuracy. 95% confidence intervals (CI) are reported for all proportions.

## Results and discussion

This work presents the first use of human-in-the-loop automated tutorial of laboratory technicians to enable a resource-limited laboratory to operate an HIVDR test with minimal training. The custom mobile application based on Aquarium operating system describes procedures and workflows for the OLA-Simple kits. Here we show that Aquarium digital guidance allowed first-time users in a small laboratory in Kenya to accurately perform the OLA-Simple kits and derive HIVDR genotypes. We also present and discuss the technicians’ feedback on their experience.

### Participant characteristics

The 12 participating technicians were 83% male, median age 30 years old (range 26–42), had a median of 6 (range 3–10) years of experience as a laboratory technician, and all were conversant in English. Their education level ranged from secondary school with a certificate in Medical Laboratory Technology to a master’s degree in a Laboratory Science, which is representative of most clinical laboratories in Kenya. They reported varied levels of experience working with HIV or molecular techniques (**Table 1**).

**Table 1:** Demographics of participants.

Variable	Value N = 12
Age, median (range), years	30 (26–42)
Gender, N (%)	
Male	10 (83.3)
Female	2 (16.7)
Primary language, N (%)	
English	4 (33.3)
Swahili	1 (8.3)
English and Swahili	7 (58.3)
Highest education level, N (%)	
Masters	1 (8.3)
Bachelor	3 (25)
Technical/Vocational training	2 (16.7)
Secondary school diploma	6 (50)
Years of experience as a lab technologist/technician, median (range)	6 (3–10)
Hours/week worked for pay in current position, median (range)	45 (40–54)
Current position in laboratory, N	
Laboratory manager	2
Lab technologist†	4
Lab technician†	6
Ever performed lab procedures related to HIV, N (%)	10 (83.3)
Viral load tests	2
CD4 testing	3
ELISA test	2
Phlebotomy and blood separation	5
Ever performed DNA or RNA extraction, N (%)	5 (41.7)
Kit (Qiagen, COBAS Ampliprep and Taqman, Abbott M2000rt)	3
Sputum lysis for GeneXpert	1
Cavidi technology	1
Ever used a thermocycler, N (%)	3 (25)
Ever set up PCR, N (%)	6 (50)

† Participants performed tests in one or several of the following areas: microbiology, hematology, biochemistry, parasitology, immunology sections, CD4/8, GeneXpert (TB), ELISA.

### Laboratory setup for Aquarium-assisted OLA-Simple training

The installation cost of OLA-Simple is low compared to automated sequencing platforms (> US$ 100,000) as it uses equipment that exists in most laboratories or is relatively inexpensive to acquire. Setting up the assay and software at the Coptic Hope Center laboratory required ~US$1,000 of additional equipment (scanner, microcentrifuge, vortexer, computer tablets, a server, and an uninterruptible power supply). For a laboratory without access to a thermal cycler, a battery-powered portable unit (~$500) can be used [23].

**Fig 1:**
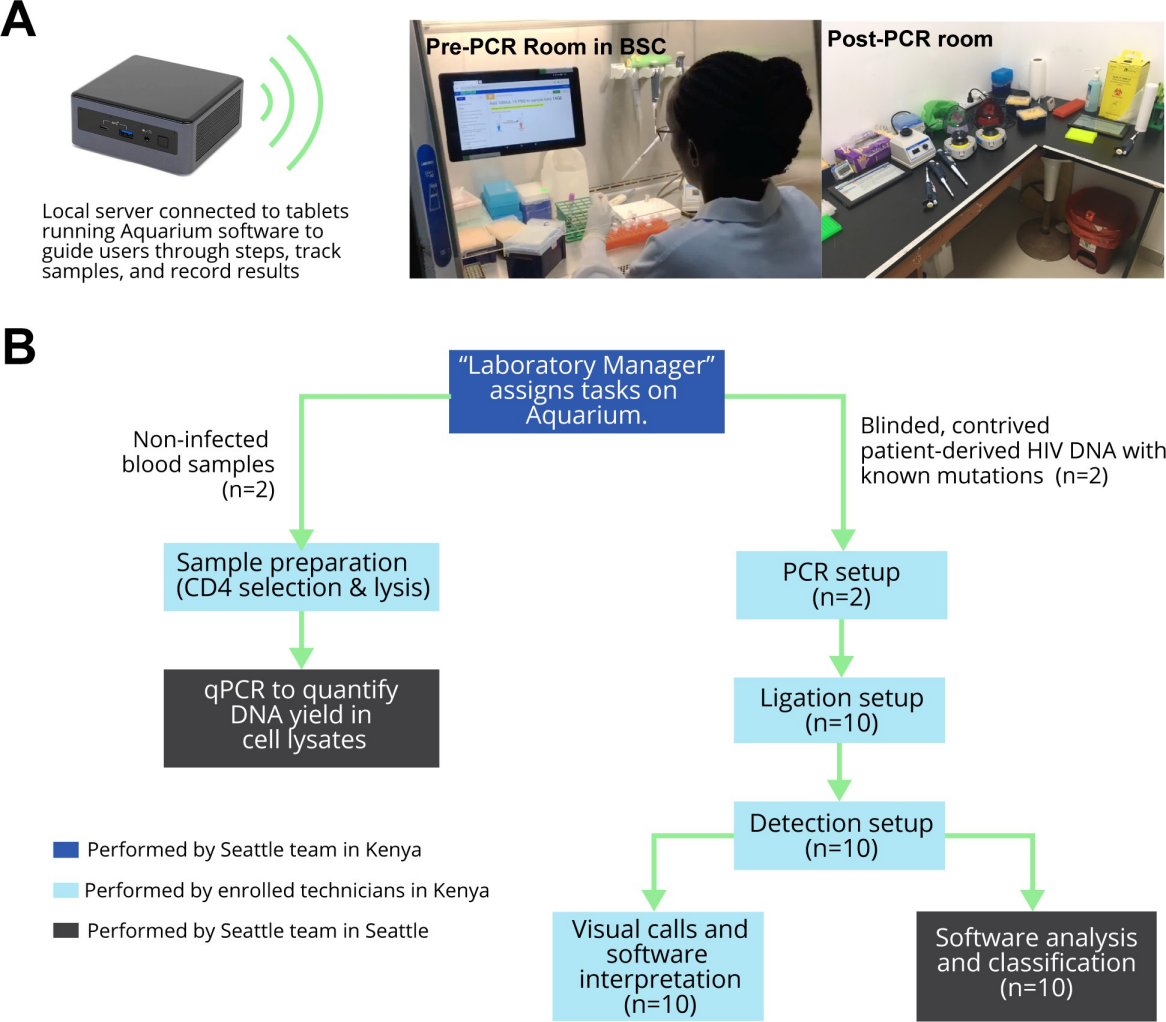
OLA-Simple laboratory setup and workflow. **(A)** Laboratory setup: Tablets were connected to a local server to run Aquarium software. The pre-PCR room had a refrigerator/freezer and a BSC where CD4+ separation and PCR reaction were set-up. Technicians controlled the Aquarium-based software on the tablet with a foot pedal (not shown in the picture) while performing CD4+ preparation. The post-PCR room had two designated bench areas to set up ligation and detection separately. This room also contained a thermal cycler and a scanner. **(B)** Tasks assigned by Aquarium for Kenyan technicians, and assessment of their performance using Aquarium-assisted OLA-Simple. The sample preparation module was separated from the amplification, ligation, detection, and interpretation module. Associated protocol code, and examples of complete runs are publicly available (https://github.com/OLA-Simple/Papers-Vrana-Panpradist-et-al-2021).

The laboratory was equipped with a Wi-Fi network to run Aquarium and coordinate assay steps across a pre-PCR and post-PCR room (**Fig 1A**). Participants worked in pairs to follow the OLA-Simple workflow (**Fig 1B**): sample preparation and PCR set-up were carried out in the pre-PCR room, while PCR, ligation and detection were conducted in the post-PCR room to minimize potential for amplicon-carryover contamination. Each participant processed two uninfected blood specimens and performed mutation testing on two contrived specimens with known HIV mutations following the interactive digital instructions provided by Aquarium.

To complete the workflow, it took each pair of participants an average of seven hours. This time included the introductory session, completion of surveys and staggering some assay steps due to space and instrument constraints. The turnaround time would likely decrease to 4.5–5 hours once users became familiar with the software and the kits.

Reported turnaround time for centralized HIVDR testing in resource-limited settings is 18 days [24] from sample collection, shipping to the laboratory, batching and testing samples, and transmission of results to the clinic. In this study, OLA-Simple was performed within the hospital grounds and thus could deliver test results within one day. Moreover, each kit tests two samples, a number suitable for the volume of daily or weekly patient samples submitted for HIVDR testing in small laboratories in Kenya, which eliminates the need to wait for an adequate number of specimens to batch for testing.

### Performance of participants using OLA-Simple

**Fig 2:**
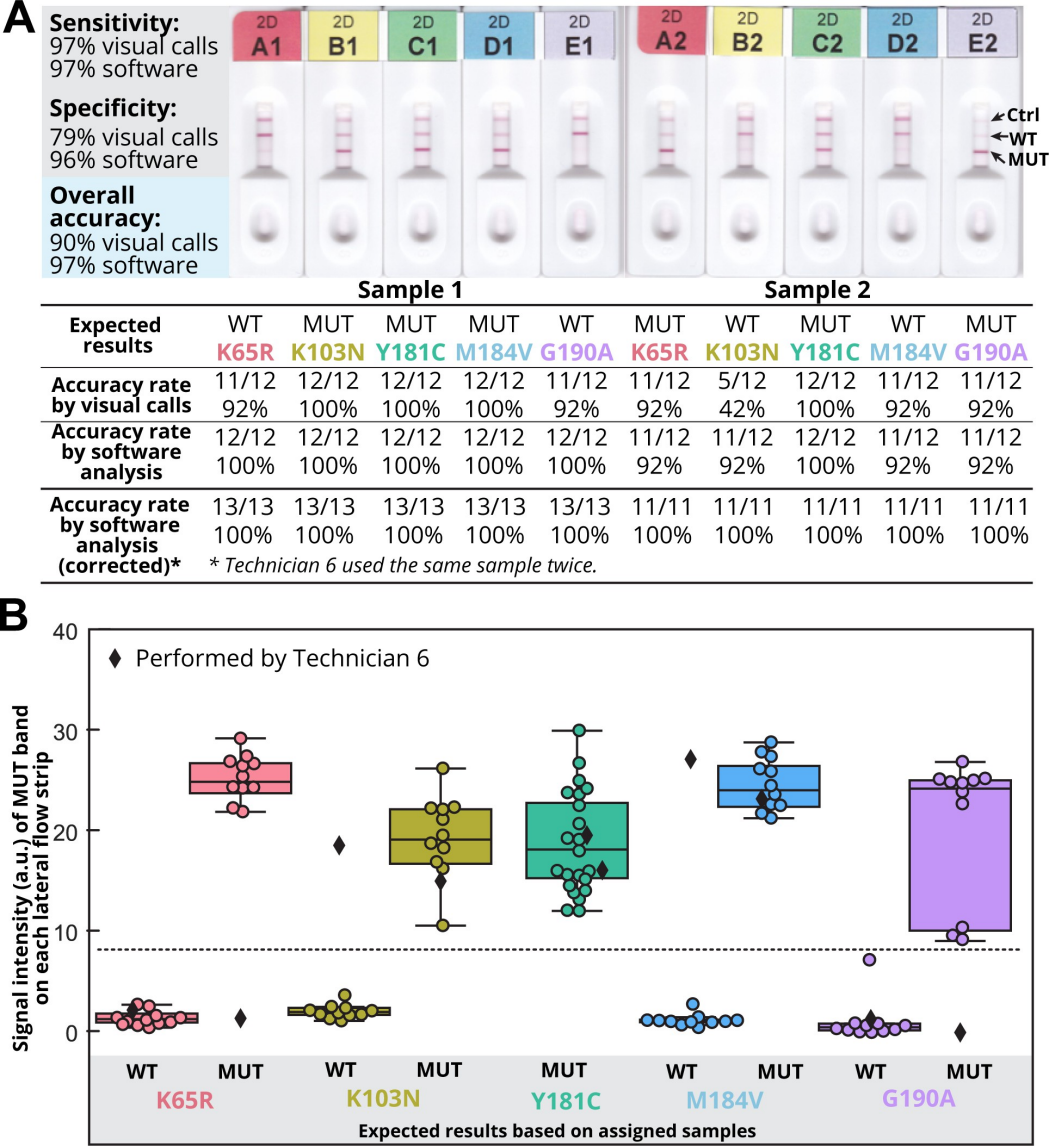
OLA-Simple HIVDR results and interpretations. **(A)** Examples of scanned images of test strips for Samples 1 and 2 and test accuracy across each codon based on visual calls made by each participant and post-processing by image analysis software. Sample1 and Sample 2 have different mutation profiles. **(B)** Mutant (MUT) signal intensity of each lateral flow strip. Middle lines on the box plot indicate medians. Top and bottom lines on box plot indicate interquartile ranges. Dashed lines indicate the detection threshold for MUT signal. Both Sample 1 and Sample 2 have mutant genotype at codon Y181C. Diamonds correspond to signal from the strips of a Sample 1 that was erroneously tested in place of Sample 2.

All 12 participants successfully isolated CD4+ cells from whole blood collected from four donors with yields within the expected range (mean±SD: 686,450±216,500 CD4+ cells/mL) and completed genotyping of two samples using the OLA-Simple kits. The two blinded DNA samples tested by all participants included wild-type genotype only or mixtures of mutant and wild-type genotypes at each of five HIV reverse transcriptase codons, including K65R, K103N, Y181C, M184V and G190A (total of 10 codons/participant) (**Fig 2A**). Participants correctly genotyped 70/72 (97.2%, 95% CI: 90–100%) mutant codons and 38/48 (79.2%, 95% CI: 65–90%) wild-type codons. The only two false negatives were due to Participant #6 erroneously testing Sample 1 twice and omitting testing of Sample 2. Of 10 false positive results, 2 were due to testing Sample 1 in place of Sample 2 by Participant #6, 1 appeared to be contamination (light mutant signal at codon G190A likely from reusing a pipette tip contaminated with mutant ligation product) and 7 were due to light mutant background at codons K103N (n = 6) and K65R (n = 1). Analysis of the scanned images using our in-house image analysis software improved test accuracy to 97% (116/120, 95% CI: 92–99%) with 97% (70/72, 95% CI: 90–99%) mutant codons and 96% (46/48 95% CI: 86–99%) wild-type codons correctly genotyped (**Fig 2B**).

The image analysis software improved accuracy over visual interpretation of results by establishing a signal threshold above the mutant background signal and thus eliminating false positives. The sample mix-up described above reduced the overall accuracy; this could be improved with changes in the kit labeling system to prevent this type of errors. Correcting for the sample that was added twice, the performance of the assay chemistry combined with software analysis yielded 100% accuracy (120/120, 95% CI: 97–100%).

### Feedback from participants

Overall, participants scored the use of software as helpful in learning to perform the assay and enjoyed the clear instructions and interpretability of the results using the tablets. They strongly agreed that they understood the meaning of the bands on the strips, and that the Aquarium instructions were easy to follow (**Table A in S1 File**). However, several participants felt the procedure was lengthy and involved too many steps (**Table B in S1 File** summarizes the survey responses). In response, we have subsequently developed new chemistries to reduce assay time to 3.5 hours by replacing the 1.5-hour blood DNA preparation with a 30-minute plasma RNA extraction and the 2-hour PCR with 1-hour RT-PCR.

Our study shows it is feasible to use Aquarium to train local laboratory personnel with basic experience in lab work and onboard an HIVDR test. However, this pilot study was limited to the use of contrived specimens to establish analytical performance and each participant was able to perform the OLA-Simple kit once. Thus, we were not able to assess the ability of each technician to maintain or improve their skills. A larger demonstration and evaluation study that includes HIV-infected clinical specimens and multiple OLA-Simple runs is ongoing in Kenya to assess the robustness of our HIVDR test.

The Aquarium software has useful features for HIVDR testing such as automatic collection of operator interactions in Aquarium’s virtual laboratory notebook that can be useful for troubleshooting. Each kit item is labeled with a unique identifier that Aquarium instructions use in conjunction with corresponding pictures to avoid ambiguity and reduce the extent of in-person training needed. Uniquely labeled items also allow tracking of stock consumption in real-time which can be useful and timesaving for laboratory management. Importantly, Aquarium could link test results to treatment algorithms to advise clinicians, and algorithms could be updated as clinical recommendations or policies change.

## Conclusion

Aquarium-based software facilitated deployment of the OLA-Simple in Kenya with minimal training, enabling local laboratory technicians to perform an HIVDR test. Human-in-the-loop automation could facilitate daily operations of laboratory-based assays and increase the performance and accuracy of diagnostic tests in small laboratories.

## Supporting information

S1 File (DOCX)
