# Supplementary material for: Implementation of an interactive mobile application to pilot a rapid assay to detect HIV drug resistance mutations in Kenya
Source: PLOS Glob Public Health. 2022 Feb 16;2(2):e0000185. doi: 10.1371/journal.pgph.0000185 (PMC10021139; doi:10.1371/journal.pgph.0000185)
Supplement: S1 File — (DOCX) [file pgph.0000185.s001.docx]

**Supporting Information. Participants’ survey responses regarding Aquarium and the OLA-Simple kit.**

Participants (N=12) were asked to fill out a short survey immediately following the procedure, which included evaluating four statements on a 5-point Likert scale (strongly agree to disagree) (**Table A**) and three open-ended qualitative questions (**Table B**). For ordinal categorical values, modes are displayed for each statement. For open-ended questions, we present a summary of the responses describing all points raised by the participants.

**Table A. Participants’ survey responses to four statements regarding Aquarium and the OLA-Simple kit**

| **Questionnaire statement** | **Mode** |
| --- | --- |
| **Specimen preparation**: I was able to perform these steps in less time than my usual DNA extraction | 4* |
| **PCR and Ligation**: Using dried reagent is easier than setting up a traditional PCR reaction | 4* |
| **Detection**: I understood the meaning of the bands in the strip | 5 |
| **Kit instructions**: Instructions were easy to follow | 5 |

* Participants 11 and 12 answered NA to the “specimen preparation” statement, and Participant 12 answered NA to the “PCR and Ligation” statement, likely due to not having performed these procedures prior to this training.

Rating scale: 1=strongly disagree, 2=disagree, 3=neutral, 4=agree, 5=strongly agree, and NA=not applicable.

**Table B**. **Summary of participants’ responses to open-ended questions regarding Aquarium and the OLA-Simple kit.**

| **What did you like best about using this kit?** | **Which instruction(s) were not easy to follow? Please explain why.** | **What advice do you have for us to make kit and instructions easier to use?** |
| --- | --- | --- |
| - The instructions were easy to follow - The protocol was easy to understand. - The examples in Aquarium were well labeled and made it easy to know where to add each reagent or sample - The kit is user-friendly and straightforward - The kit is easy to use compared to the plate-based OLA method - There was only one PCR step instead of the usual two for nested PCR - Detection was simplified by Aquarium - Results are generated fast and are also portable - It is easy to read the bands and interpret the results: it clearly shows the control, the wild-type and the mutant - A single sample can be easily processed without the need for batching | - The instructions and procedure were clear and easy to follow. - The instructions were Ok when following a step, but it was difficult to memorize the steps without a written protocol to follow - Some instructions did not include the specific vial or reagent in the heading, but it was in the pictorial - Sample preparation: - the multiple tubes and SOP instructions were not alphabetically arranged - includes many tubes, color coding would help to identify which to use first and avoid mix-ups - too many steps make it confusing - the process is long with many reagents and waiting time - During the ligation/detection step had challenges remembering to add reagent to the next strip - At times the tablet was not working, and we did not have a backup plan such as a written protocol with instructions. | - Enlarge the font under every heading. - Include the reagent vial ID on the headings for better clarity - The tube labels should match the SOP instruction labels. - Harmonize the sequence of adding reagents and closing of tubes to reduce multitasking and possible confusion. - Create system for how to keep track of addition of reagents to tubes - State what to expect at the end of each step, e.g. maybe a clean supernatant /deposit so one can confidently move to the next step - Look for alternatives to replace the current sample preparation method - Make the incubation time shorter - Improve turn-around time for clients. - Have print outs of the protocols as a back-up plan. - Make the kit commercially available with all the steps for laboratory technologists to follow. |
